# Remarkable diversity of intron-1 of the *para* voltage-gated sodium channel gene in an *Anopheles gambiae*/*Anopheles coluzzii* hybrid zone

**DOI:** 10.1186/s12936-014-0522-1

**Published:** 2015-01-21

**Authors:** Federica Santolamazza, Beniamino Caputo, Davis C Nwakanma, Caterina Fanello, Vincenzo Petrarca, David J Conway, David Weetman, Joao Pinto, Emiliano Mancini, Alessandra della Torre

**Affiliations:** Dipartimento di Sanità Pubblica e Malattie Infettive, Istituto Pasteur-Fondazione Cenci-Bolognetti, Università “Sapienza”, Piazzale Aldo Moro 5, 00185 Rome, Italy; Medical Research Council Unit, Fajara, P.O. Box 273, Banjul The Gambia; Centre for Tropical Medicine, Nuffield Department of Medicine, University of Oxford, Oxford, UK; Dipartimento di Biologia e Biotecnologie, Istituto Pasteur-Fondazione Cenci-Bolognetti, Università “Sapienza”, Piazzale Aldo Moro 5, 00185 Rome, Italy; Vector Biology Department, Liverpool School of Tropical Medicine, Liverpool, UK; Centro de Malária e outras Doenças Tropicais, Instituto de Higiene e Medicina Tropical, Universidade Nova de Lisboa, Lisbon, Portugal; Dipartimento di Scienze, Università Roma Tre, Viale Marconi 446, 00146 Rome, Italy

**Keywords:** Mosquito, Malaria, Hybridization

## Abstract

**Background:**

Genomic differentiation between *Anopheles gambiae* and *Anopheles coluzzii* - the major malaria vectors in sub-Saharan Africa - is localized into large “islands” toward the centromeres of chromosome-X and the two autosomes. Linkage disequilibrium between these genomic islands was first detected between species-specific polymorphisms within ribosomal DNA genes (IGS-rDNA) on the X-chromosome and a single variant at position 702 of intron 1 (Int-1^702^) of the *para* Voltage-Gated Sodium Channel (VGSC) gene on chromosome arm 2 L. Intron-1 sequence data from West and Central Africa revealed two clearly distinct and species-specific haplogroups, each characterized by very low polymorphism, which has been attributed to a selective sweep. The aim of this study was to analyse Int-1 sequence diversity in *A. gambiae* and *A. coluzzii* populations from the Far-West of their range, in order to assess whether this selective-sweep signature could persist in a zone of high interspecific hybridization.

**Methods:**

A 531 bp region of VGSC Int-1 was sequenced in 21 *A. coluzzii*, 31 *A. gambiae*, and 12 hybrids from The Gambia and Guinea Bissau, located within the Far-West geographical region, and in 53 *A. gambiae s.l.* samples from the rest of the range.

**Results:**

Far-West samples exhibit dramatic Int-1 polymorphism, far higher within each country than observed throughout the rest of the species range. Moreover, patterning of haplotypes within *A. coluzzii* confirms previous evidence of a macro-geographic subdivision into a West and a Central African genetic cluster, and reveals a possible genetic distinction of *A. coluzzii* populations from the Far-West.

**Conclusions:**

The results suggest a relaxation of selective pressures acting across the VGSC gene region in the hybrid zone. Genetic differentiation in the Far-West could be attributable to a founder effect within *A. coluzzii*, with subsequent extensive gene flow with secondarily-colonizing *A. gambiae*, potentially yielding a novel insight on the dynamic processes impacting genetic divergence of these key malaria vectors.

**Electronic supplementary material:**

The online version of this article (doi:10.1186/s12936-014-0522-1) contains supplementary material, which is available to authorized users.

## Background

Mosquito species belonging to the Afro-tropical *Anopheles gambiae* complex represent a valuable model for studies of ecological speciation (‘speciation with gene flow’) [1,2]. In fact, owing to the major role of some of these species in malaria transmission, their genetic divergence has been studied extensively for more than half century, revealing repeated events of ‘ecotypic speciation’ [3,4]. These studies have revealed the existence of morphologically indistinguishable, but chromosomally/genetically distinct species with very different roles as malaria vectors (*A. gambiae sensu stricto, Anopheles arabiensis, Anopheles quadriannulatus, Anopheles amharicus, Anopheles melas, Anopheles merus, Anopheles bwambae*) [5,6]. In addition to these sibling species, which are isolated by both pre-and post-mating mechanisms, more recent studies have highlighted a further subdivision within the nominal species *A. gambiae s.s.*, the most synanthropic and efficient malaria vector of the complex [7,8]. These studies have revealed the existence of two taxonomic units initially named the ‘M’ and ‘S’ molecular forms and now formally raised to species as *Anopheles coluzzii* and *A. gambiae s.s.* (hereafter referred as *A. gambiae*) [9], respectively. The two species are isolated only by (partially understood) pre-mating mechanisms and show limited genomic differentiation, which is localized most prominently in low-recombination peri-centromeric regions of chromosome-X and of the two autosomes [10-13]. These regions have been postulated to represent ‘genomic islands of speciation’, expanding in size by selection across linked loci connected to reproductive isolation, as predicted by ecological speciation with gene flow models [1,2]. However, their large size is probably enhanced by locally reduced recombination [10,14,15], leading to suggestions that high centromeric differentiation is primarily a result of recurrent background selection and hitchhiking unrelated to speciation [2,16]. Interestingly, the species-specific linkage disequilibrium among the three physically-unlinked centromeric regions is widespread [13], but has been lost at the western extreme of their range (*i.e.* the ‘Far-West’, from The Gambia to Guinea Bissau). In this putative secondary contact zone between the two species, high frequencies of hybrids are found and pronounced inter-specific differentiation is maintained only on the chromosome-X centromere [17-21].

The first evidence of genetic linkage between the chromosome-X and chromosome-2 genomic island regions came from pre-genomic studies showing that the SNPs within the ribosomal intergenic spacer region (IGS- rDNA), which define the two species [7], are in strong linkage disequilibrium (LD) with a SNP at position 702 of Intron-1 (hereafter named Int-1^702^) of the *para* Voltage-Gated Sodium Channel (VGSC) gene [22,23]. The VGSC gene has been studied extensively because of the presence of two mutations in the exon immediately downstream of Int-1, causing a change from Leucine to Serine (L1014S) or to Phenylalanine (L1014F). Each mutation can confer *knock-down resistance* (*kdr*) to DDT and pyrethroid insecticides [22,23]. In West and Central Africa the Int-1^702^ SNP, which exhibits diagnostic nucleotides for each species (*i.e. A. coluzzii* = Int-1^C^; *A. gambiae* = Int-1^T^), defines two clearly distinct and species-specific haplotype groups each characterized by very low polymorphism. This led to the hypothesis of a selective sweep, pre-dating *kdr* mutations, centred on favourable variants in nearby genes that might contribute to the segregation of the two species [23].

The aim of this study was to investigate sequence diversity of the Int-1 region of the VGSC in *A. gambiae* and *A. coluzzii* populations from the Far-West to assess whether the selective-sweep observed in the rest of the species range is maintained in the face of high interspecific gene flow.

## Methods

### *Anopheles gambiae sensu lato* sample identification and *kdr* genotyping

*Anopheles gambiae s.l.* specimens were identified as *A. arabiensis*, *A. quadriannulatus*, *A. melas* or *A. merus* using the method of Scott *et al.* [24], and as *A. coluzzii* or *A. gambiae s.s.* by using both PCR-RFLP of the intergenic spacer (IGS) rDNA region [25] and the *SINE*-PCR [26] methods. The latter method is based on an *A. coluzzii-*specific and irreversible single-locus insertion of a *SINE200* retrotransposon in the X-chromosome centromeric region, about 1.5 Mb from IGS region containing *A. coluzzii vs A. gambiae* species-specific SNPs. The *kdr* 1014 locus was genotyped by either allele-specific-PCR (AS-PCR) or Hot Oligonucleotide Ligation Assay (HOLA) methods [27-29] in *A. coluzzii* and *A. gambiae*. Genotypes of *kdr* 1014 position in *A. arabiensis*, *A. quadriannulatus, A. melas* and *A. merus* were obtained by direct sequencing of a 531 bp fragment [27].

### Sequencing of Int-1 of VGSC gene

Amplification of a 531 bp region of Int-1 of the VGSC gene (AGAP004707 in genome build AgamP4.1) was carried out using the “Ganest” primer (5′- CAT ACA TTG CTT AAA GCT CTA ATT ATC -3′), located upstream at positions 388–414 in the Int-1 region, coupled with the “Montrev” primer (5′- CAC AAG GCA CAC GAT ACG -3′), located downstream at positions 995–1013 at the end of intron-2 (nucleotide positions as in [22]). The PCR mixture contained: 10× PCR Buffer (Bioline), 2 mM MgCl_2_, 200 μM dNTPs equimolar mix, 1 U Taq DNA polymerase (Bioline) and 0.25 μM of each primer, in a total volume of 25 μl. Cycling conditions were as follows: 94°C for 5 min., 35 cycles each with 94°C for 30 sec./ 50°C for 35 sec./ 72°C for 1 min., followed by a final extension step of 10 min at 72°C. Direct sequencing was performed according to [22] and [23].

Chromatograms were edited using the Staden Package ver. 2003.1.6 [30] and sequences were aligned using MAFFT ver. 5 [31]. Haplotype inference was performed with the PHASE algorithm [32] as implemented in DnaSP v5.10.01 [33]. Haplotype sequences reported in Gentile *et al.* [23] were also included for comparison. Sequences were deposited in Genbank under accession n° KP300645-KP300752.

### Data analysis

DnaSP v. 5.10.01 [33] was used to produce estimates of Int-1 polymorphism and perform Tajima’s *D* and Fu and Li’s D* and F* [34,35] neutrality tests. Genealogical relationships among Int-1 haplotypes were reconstructed by computing parsimony networks using TCS 1.21 [36]. Haplotypes names were retained for those previously reported in [23], while novel M1- and S1-related haplotypes were either named with consecutive numbers or with specific codes when exclusive of the Far-West region (*i.e.* GU for Guinea Bissau, GA for The Gambia, and GUGA for both countries). Frequency and distribution of Int-1 haplotypes in the Far West, West and Central African geographic regions in *A. coluzzii*, *A. gambiae* and putative hybrids (by merging original and previous data from [23]) were also computed. Inter- and intra-specific *Fst* estimates of genetic differentiation were calculated using Arlequin 3.11 [37].

## Results

A 531 bp region of the VGSC gene Int-1 was sequenced in 21 *A. coluzzii*, 31 *A. gambiae*, 12 *A. gambiae* x *A. coluzzii* hybrids from the Far-West region. These sequences were aligned with those obtained by Gentile *et al.* [23] and with 53 additional original sequences from *A. coluzzii* and *A. gambiae* populations from the rest of the distribution range, as well as from other species of the complex (Additional file 1: Table S1), resulting in a total of 490 sequences. Note that *A. coluzzii* and *A. gambiae* were identified by both the IGS marker and *SINE* insertion, which provided completely consistent identification in all samples, with the notable exceptions of those from The Gambia (55% inconsistent) and Guinea Bissau (51% inconsistent). Inconsistently identified individuals were classified based on the *SINE* marker, as this is not biased toward the hybrid genotype by intra-chromosome recombination known to occur in the IGS multicopy rDNA region [38]. Specimens were genotyped for *kdr*-mutations: all carried the wild type/insecticide *kds* susceptible allele of VGSC gene (*i.e.* TTA) with the exception of two out of eight individuals from Rwanda carrying the TCA allele (hereafter L1014S) in homozygosis and three carrying the TTT allele (hereafter L1014F) in heterozygosis (Additional file 2: Table S2).

**Figure 1:**
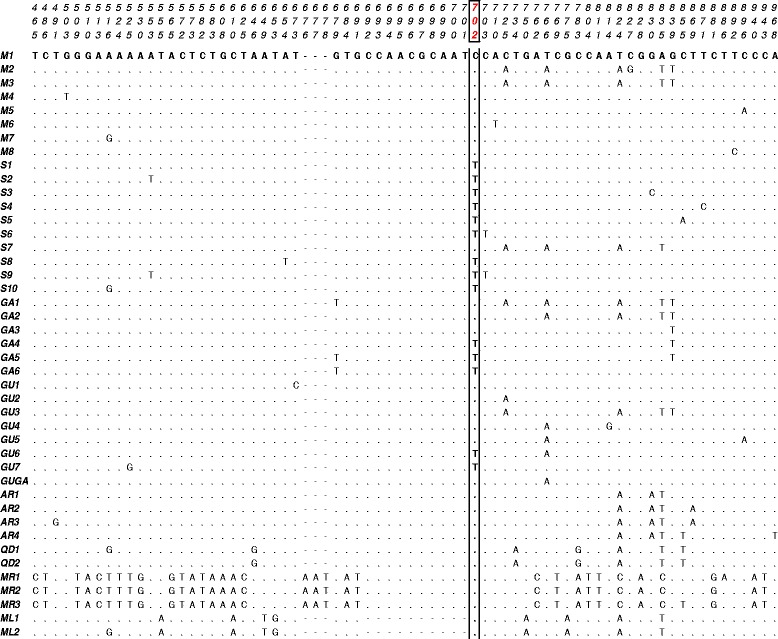
**VGSC intron-1 (Int-1) polymorphism within the** ***Anopheles gambiae*** **complex.** Nucleotide alignments show variable positions among Int-1 haplotypes. Positions are numbered as in Gentile *et al.* [23] and site 702 (*i.e.* Int-1^702^) is highlighted in red. Haplotypes are named as follows: M1-M6 and S1-S6 = *Anopheles coluzzii* and *A. gambiae* haplotypes as in Gentile *et al.* [23]; M7-M8 and S7-S10 are novel *A. coluzzii* and *A. gambiae* haplotypes not exclusive to The Gambia and Guinea Bissau; GU, GA or GUGA = *A. coluzzii* and *A. gambiae* private haplotypes from either Guinea Bissau, The Gambia, or both Countries, respectively. AR1-AR4 = *Anopheles arabiensis* haplotypes; QD1-QD2 = *Anopheles quadriannulatus* haplotypes; MR1-MR3 = *Anopheles merus* haplotypes; ML1-ML2 = *Anopheles melas* haplotypes.

**Figure 2:**
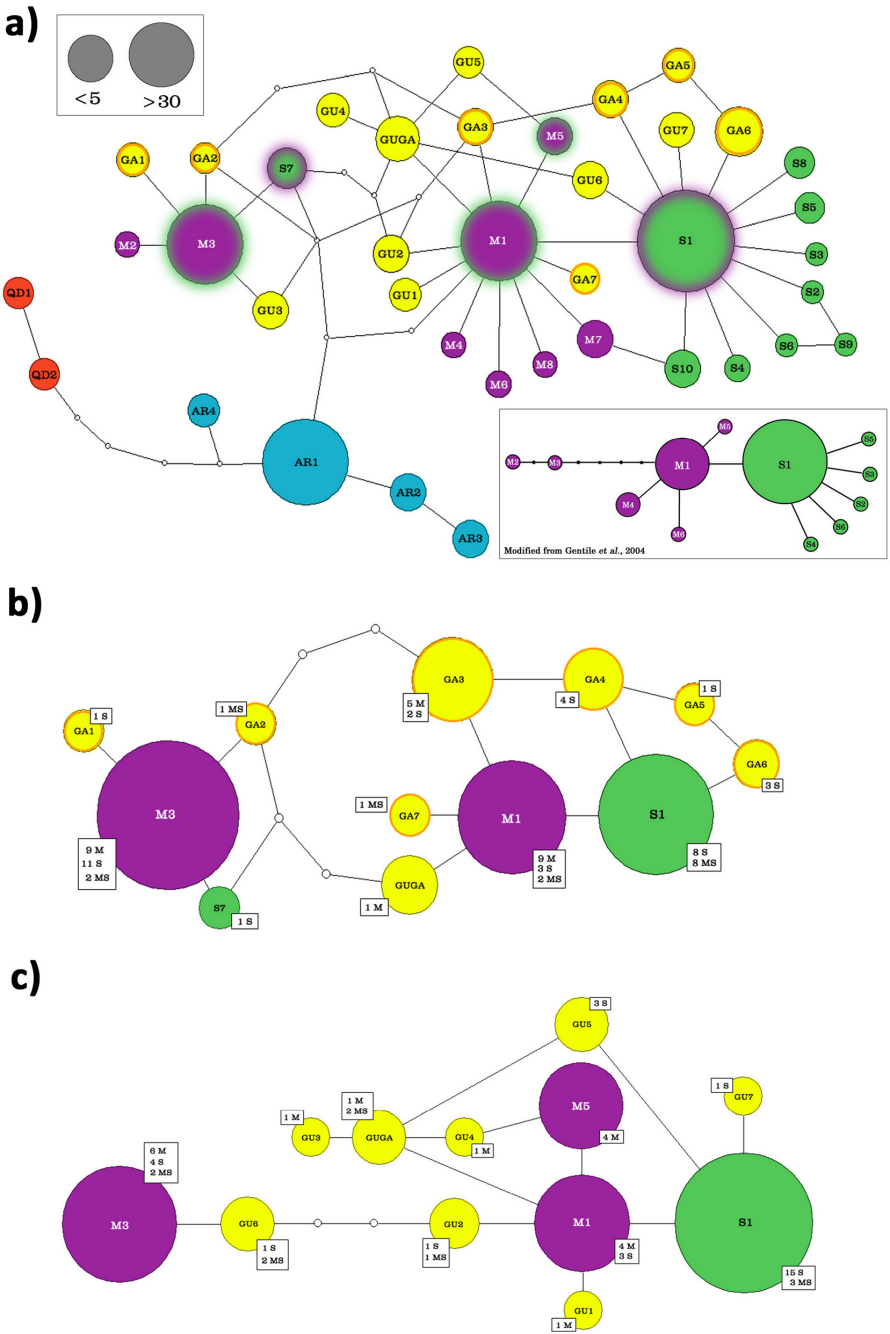
**Parsimony-based networks of genealogical relationships among VGSC Intron-1 haplotypes in the** ***Anopheles gambiae*** **complex. a)** Network built with original data collected from all studied species of the *A. gambiae* complex, with the exception of *Anopheles melas* and *Anopheles merus*, whose haplotypes exceeded the 95% threshold of TCS connection limit. Haplotypes are represented by pies whose sizes are proportional to frequencies in the sample and coloured as follows: blue = *Anopheles arabiensis* (AR), red = *Anopheles quadriannulatus* (QD), violet = *Anopheles coluzzii*, green = *Anopheles gambiae* (haplotypes are named and numbered according to Gentile *et al.* [23]), yellow = private haplotypes from either Guinea Bissau (GU), The Gambia (GA), or from both Countries (GUGA); sequential codes are used to name *A. coluzzii* (*i.e.* M7, M8) and *A. gambiae* (S7-S10) novel haplotypes not exclusive to The Gambia and Guinea Bissau; M1, M3, M5, S1 and S7, which are not completely segregated between the two species in the Far-West and/or Rwanda, are shaded. Below: VGSC Int-1 network for M (*A. coluzzii*) and S (*A. gambiae*) molecular forms as in Gentile *et al.* [23]; **b)** and **c)** networks only including *A. coluzzii, A. gambiae* and hybrids haplotypes from The Gambia and Guinea Bissau, respectively. White squares report numbers of alleles for *A. coluzzii* (M), *A. gambiae* (S) and hybrids (MS) (identified based on *SINE*-PCR [26]) included in each haplotype.

Variable sites among haplotypes, and their relationships among different members of the *A. gambiae* complex, are shown in Figure 1 and in Figure 2, respectively. All species are well-separated from each other and from *A. coluzzii* and *A. gambiae* (except for one *A. arabiensis* individual from Senegal sharing the S1-haplotype with *A. gambiae*). Interestingly, *A. gambiae* carries a C to T mutation at site 702 (hereafter Int-1^702^, in red in Figure 1) separating it from all other species. This mutation was already shown by Gentile *et al.* [23] to separate “S-molecular form-S1 (Int-1^T^)” from “M-form-M1 (Int-1^C^)” haplotypes and closely-related locale-specific variants. In the Gambian and Guinean samples, however, *A. coluzzii*-specific Int-1^C^ haplotypes are also found in *A. gambiae*, but no *A. gambiae*-specific Int-1^T^ haplotypes are found in *A. coluzzii* (Figures 2b and 2c; Additional file 2: Table S2). This result does not change if the species are identified using the IGS marker rather than by *SINE*-PCR, as presented so far.

**Figure 3:**
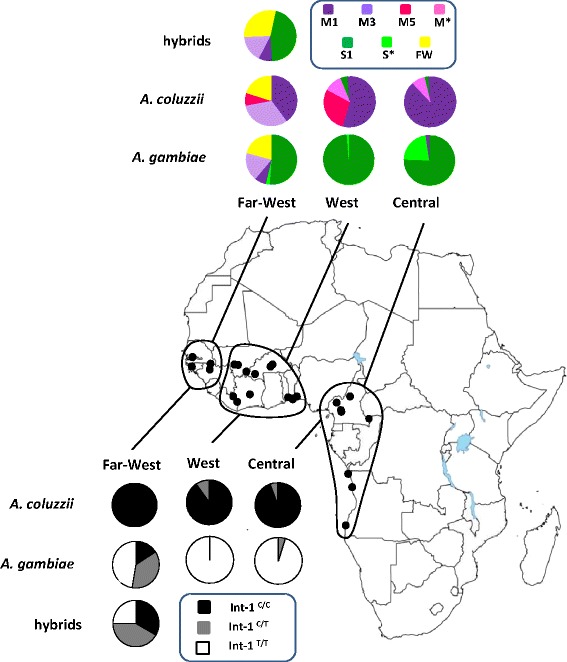
**Frequency and distribution of VGSC Intron-1 haplotypes and of Int-1** ^**702**^ **genotypes in** ***Anopheles gambiae*** **,** ***Anopheles coluzzii*** **and hybrids.** VGSC Int-1 haplotypes M1, M3, M5 (dark violet) and S1 (dark green) are named as in Figure 1, M* = sum of all other ‘M1’-related haplotypes (pink), S* = sum of all other ‘S1’-related haplotypes (light green), “FW” = sum of all Far-West private haplotypes (yellow). Int-1^C/C^ (black), Int-1^T/T^ (white) and Int-1^C/T^ (grey) identify genotypes at site 702 (Int-1^702^). Dots on the Africa map correspond to sampling localities reported in Additional file 1: Table S1 and are grouped as follows: Far-West (N = 156) = Senegal, Gambia, Guinea Bissau, Guinea Conakry; West (N = 172) = Mali, Ivory Coast, Burkina Faso, Benin, Nigeria; Central (N = 82) = Cameroon and Angola. Data include original sequences and those from Gentile *et al.* [23].

Overall, Int-1 haplotypes are not uniformly distributed across the ranges of *A. gambiae* and *A. coluzzii* (Figure 3). Populations from the Far-West region (*i.e.* Senegal, The Gambia, Guinea Bissau, Guinea Conakry) differ from all others by the exclusive presence of the M3 haplotype (freq = 18% in *A. gambiae*, 32% in *A. coluzzii* and 16% in hybrids). Moreover, populations from The Gambia and Guinea Bissau possess 15 exclusive haplotypes (freq = 20% in both *A. gambiae* and *A. coluzzii*, 29% in hybrids), highlighted in yellow in Figure 2. M1 is the most frequent haplotype in both West and in Central African *A. coluzzii* populations, while the M5 haplotype reaches frequencies up to 30% in West African samples and is absent in Central ones. Haplotype S1 is almost the sole haplotype found in western *A. gambiae* populations (Mali, Ivory Coast, Burkina Faso and Nigeria; freq = 99%), whereas S1-related haplotypes are much more frequent in central ones (*i.e.* Cameroon and Angola; freq. = 22%). Finally, both S1 and the *A. coluzzii*-specific M1 haplotype are found at comparable frequencies (nearly 50% each) in *A. gambiae* from Rwanda (Additional file 2: Table S2).

**Figure 4:**
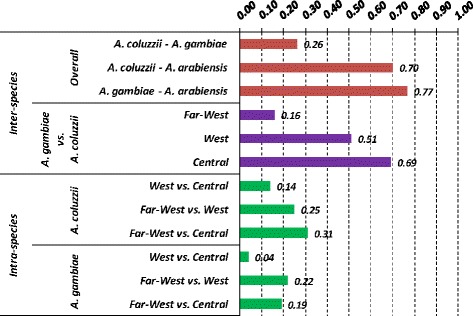
**Inter- and intra-specific** ***Fst*** **values based on VGSC Intron-1 sequence data.** Above: inter-specific *Fst* values among *Anopheles gambiae*, *Anopheles coluzzii* and *Anopheles arabiensis* (red) and between *A. gambiae* and *A. coluzzii* in each geographic region (violet). Below: intra-specific *Fst* values among populations from different geographic regions within *A. coluzzii* and *A. gambiae* (green). *Fst* are all significant (*p* < 0.05). Data include original sequences and those from Gentile *et al.* [23].

Differentiation between *A. coluzzii* and *A. gambiae* in Central Africa (*Fst* = 0.69) is comparable to that observed between each species and *A. arabiensis* (*Fst* = 0.70 and 0.77, respectively), but decreases westwards (*Fst* in West-Africa = 0.51 and in Far-West region = 0.16) (Figure 4). Intraspecific *Fst* values are slightly lower between West and Central populations of each species (*Fst* = 0.04 and 0.14) than between Far-West samples and West and Central ones (*Fst* ranging from 0.19 to 0.31) (Figure 4).

Table 1 shows genetic diversity and summary statistics for *A. coluzzii* and *A. gambiae* samples from Far-West, West, Central Africa, as well as from Rwanda, including original data and those from West/Central Africa reported by Gentile *et al.* [23]. In general, nucleotide/haplotype diversity is low in samples from West and Central Africa (π = 0.02-0.21), but increases in the Far-West region (π = 0.47-0.49). Statistics applied to detect departures from neutrality reveal a weak trend toward positive values in *A. gambiae* and *A. coluzzii* (and hybrids) from “Far-West”, whereas negative values were scored in both species from West and Central Africa (significant in *A. gambiae*: D* = −2.583, F* = −2.686, and *A. coluzzi*: D* = −2.848, F* = −2.922) as well as in *A. gambiae* from Rwanda, suggesting the influence of different selective pressures and/or demographic histories in populations from different geographic areas.

**Table 1:** **Intron-1 of VGSC gene polymorphism and summary statistics in** ***Anopheles coluzzii*** **and** ***A. gambiae*** **samples**

	**African region**	**n**	**H**	**Hd**	**S**	**π (%)**	**θ (%)**	***Tajima D***	**Fu & Li’s** ***D***	**Fu & Li’s** ***F***
***A. coluzzii***										
	**Far-West**	50	8	0.73	8	0.49	0.34	1.217	−0.127	0.361
	**West**	92	7	0.68	11	0.21	0.41	−1.303	0.120	−0.450
	**Central**	38	4	0.15	3	0.03	0.14	−1.720	−2.848*	−2.922*
***A. gambiae***										
	**Far-West**	82	14	0.70	9	0.47	0.34	0.926	−0.123	0.277
	**West**	78	3	0.12	2	0.02	0.08	−1.121	−1.011	−1.218
	**Central**	46	8	0.51	7	0.12	0.30	−1.657	−2.583*	−2.686*
	**East**	16	4	0.65	6	0.22	0.34	−1.230	−2.025	−2.076
**Hybrids**										
	**Far-West**	24	8	0.77	7	0.52	0.36	1.472	0.629	1.016

Data include original sequences and those from Gentile *et al.* [23]. Far-West = Senegal, Gambia, Guinea Bissau, Guinea Conakry; West = Mali, Ivory Coast, Burkina Faso, Benin, Nigeria; Central = Cameroon and Angola; East = Rwanda. n = n° alleles; H = n° haplotypes; S = n° segregating sites; Hd = haplotype diversity; π = nucleotide diversity; θ = Watterson estimate of theta; for Tajima *D*, Fu & Li’s *D* and *F* values, *p* computed using confidence levels provided by the coalescent (DNAsp 5.0) * < 0.05.

## Discussion

Data on Int-1 of the VGSC gene highlight dramatic genetic differences between *A. coluzzii* and *A. gambiae* populations from West and Central Africa and those from the western extreme of the species range, where inter-specific gene-flow is elevated and inter-specific differentiation reduced.

In West and Central Africa, Int-1 genetic differentiation between *A. coluzzii* and *A. gambiae* is high and comparable to that observed in their sibling *A. arabiensis* (Figure 4). Across this wide geographical region, as previously shown [23], the two species exhibit low nucleotide/haplotype diversity and are strongly segregated based on two main Int-1 haplotypes (*A. coluzzii*-M1 and *A. gambiae*-S1, separated by a single C-T mutational step at site Int-1^702^) and species-specific rare variants stemming from these. To explain this low polymorphism, a selective sweep centered on a favourable variant in a nearby gene was suggested [22,23]. Since the VGSC gene includes mutations conferring *kdr* resistance to insecticides, a possible hypothesis is that Int-1 haplotypes are in linkage with these strongly selected alleles. Introgression of *kdr* alleles and adjacent genomic regions has repeatedly been reported from different geographic areas [19,39]. Indeed, whole genome sequence data have recently shown that over 3 Mb has introgressed from *A. gambiae* to *A. coluzzii* in Ghana along with the *kdr* L1014F mutation [40]. It is possible that selection on *kdr*-associated Int-1 haplotypes may have had a role in reducing diversity, as *kdr*-resistance has been reported in some of the *A. gambiae* analysed populations particularly from West Africa [41,42] [but not in the Far-West region, Pinto *et al.*, unpublished observations; see discussion below]. However, the close physical proximity of *kdr* and Int-1 would make recombination between resistant and susceptible *kdr* alleles and their linked Int-1 polymorphisms highly unlikely over a short timescale. Moreover, in *A. coluzzii*, as the samples analyzed came from populations where *kdr*-alleles were either absent or present at moderate to low frequencies (*i.e.* populations from Benin, 40.0%, Nigeria, 19.5% and Cameroon, 6.3% [42]), and are thus likely to have introgressed very recently [19,22,39]. Recent genomic studies have given additional hints for understanding the reduction in genetic variation at Int-1 and its linkage disequilibrium with markers on the physically unlinked X-centromeric region defining the two species. In fact, as already mentioned, the VGSC gene is located within the chromosome-2 “genomic island” of highest divergence between *A. coluzzii* and *A. gambiae*. Under the “speciation island” [10] scenario, it can be hypothesized that a hitchhiking effect on Int-1 has occurred due to diversifying selection on a chromosome-2 “island” gene participating in the building-up of pre-mating barriers, or conferring differential ecological adaptation and niche segregation between *A. gambiae* and *A. coluzzii.* Alternatively, under the “incidental island” scenario [2,13,16], substitutions at Int-1^702^ may have become fixed after species splitting and accumulated little genetic differentiation due to reduced recombination in the chromosome-2 centromeric region. Interestingly, however, the association between chromosome-X and −2 “islands” is neither observed in Rwanda (Additional file 2: Table S2) nor in Tanzania [41]. In these East African sites, both M1 and S1 haplotypes were found segregating in *A. gambiae* populations. Thus, if Int-1^C^ represents the ancestral allele in the *A. gambiae* complex (Figure 1), then Int-1^C/T^ may be considered an ancestral polymorphism retained in *A. gambiae* populations from East Africa (where *A. coluzzii* is absent), which became fixed in westward sympatric areas after the splitting of the two species.

In the Far-West region, a strong reduction of inter-specific genetic divergence between *A. gambiae* and *A. coluzzii* is found - as indicated by the lower F*st* observed in this region (0.14) as opposed to the rest of the range (0.51-0.69) (Figure 4) - and a preferential introgression of M1-related Int-1 haplotypes (“typical” of *A. coluzzii*) into *A. gambiae* is observed. These data are consistent with previous studies showing weak association between chromosome-X and −2 centromeric regions and occurrence of asymmetric introgression from *A. coluzzii* into *A. gambiae* in the westernmost extreme of their range [17-21]. Furthermore, 15 exclusive Far-West haplotypes were inferred through PHASE [32] and found interspersed and connected to M1 and S1 geographically widespread variants and to the Far-West-specific M3 (Figure 2). The presence of such private haplotypes might indicate that selective pressures on the chromosome-2 centromere observed in Central African populations (Table 1) are relaxed in the Far-West. Note that, although recombination along the centromeric 500-bp Int-1 fragment analyzed would normally be considered minimal, some reduction in accuracy might occur when reconstructing haplotypes using the PHASE algorithm in the Far-West region, where LD along the 2 L-centromere is known to be lower than in the rest of the species range [19]. However, the PHASE results are supported by summary statistics (Table 1) also indicating extreme Int-1 diversity and recent introgression events in Far-West populations of both species.

There are contrasting possible explanations for the remarkable Int-1 polymorphism observed in the Far-West region. Under the *‘speciation island’* hypothesis [10] relaxation of diversifying selection on a key isolating trait on chromosome-2 centromeric “island” (of which Int-1 is a part) may have contributed to weaken pre-mating barriers between *A. gambiae* and *A. coluzzii* and promote a higher rate of gene-flow in the Far-West region. This hypothesis, however, is in contrast with data from other West and Central African areas, where introgression from *A. gambiae* to *A. coluzzii* of a *kdr*-related genomic portion in linkage with Int-1 does not produce an increase in hybridization rates [21]. Alternatively, the genomic region linked to Int-1 may be not related to speciation [2,16] and the observed pattern in the Far-West region could be attributed to a relaxation of purifying selection operating separately within each species on adaptive genetic traits not directly (or only weakly) involved in reproductive isolation. Hence, following this hypothesis, increased Int-1 polymorphism in the “Far-West” region might be the consequence of an increased recombination rate within the 2 L-centromeric “island” (and Int-1) following disruption of linked (background) selection. Resolution of these competing hypotheses requires assessment of the role of hybridization on the extent of linkage disruption throughout the 2 L-“island” and understanding of whether and how this might affect association with traits critical to speciation.

Finally, the frequency and distribution of Int-1 haplotypes within *A. coluzzii* across its range provides some hints on further intra-specific geographical patterns (Figure 3). In fact, populations from the West and Far-West regions are characterized by the exclusive presence of haplotype M5, not observed in those from Central Africa. This is consistent with results obtained by other nuclear markers (e.g. microsatellites) showing a macro-geographic subdivision into two distinct West and Central African genetic clusters, corresponding to the forest-savannah biome transition, which may have acted as an ecological barrier to gene flow [26,43,44]. Moreover, the high frequency of the Far-West exclusive M3-haplotype (separated from the major and widespread M1-haplotype by 5 mutational steps) allows speculation that a founder effect (followed by either selection or drift) affected *A. coluzzii* populations colonizing this region in the past. This last point merits further investigation through a multi-locus approach at a wider genome scale to shed light on the genetic characteristics of source populations originating the *A. gambiae*/*A. coluzzii* hybrid zone.

## Additional files

Additional file 1: Table S1.
*Anopheles gambiae* complex sample for Intron-1 of VGSC gene sequencing data. GA *= A. gambiae*; CO = *Anopheles coluzzii*; H = putative *A. coluzzii* x *A.gambiae* hybrids; AR = *Anopheles arabiensis*; QD = *Anopheles quadriannulatus*; ML = *Anopheles melas*; MR = *Anopheles merus*. CO, GA and H are defined based on *SINE*-PCR (Santolamazza *et al.*, 2008). Additional file 2: Table S2. Intron-1 genotypes in *Anopheles coluzzii* and *Anopheles gambiae* (and putative hybrids) sampled in this study. N = number of genotyped individuals; Int-1^702^ = intron-1 genotype at position 702 (following Gentile *et al.*, 2004); *kdr* = knock-down mutation at residue 1014:^§^ TTA = 1014 L (wild type); TTT = 1014 F (*kdr*-West); TCA = 1014S (*kdr*-East).
