# Supplementary material for: Remarkable diversity of intron-1 of the para voltage-gated sodium channel gene in an Anopheles gambiae/Anopheles coluzzii hybrid zone
Source: Malar J. 2015 Jan 21;14:9. doi: 10.1186/s12936-014-0522-1 (PMC4308935; doi:10.1186/s12936-014-0522-1)
Supplement: Additional file 1: Table S1. — Anopheles gambiae complex sample for Intron-1 of VGSC gene sequencing data. GA = A. gambiae; CO = Anopheles coluzzii; H = putative A. coluzzii x A.gambiae hybrids; AR = Anopheles arabiensis; QD = Anopheles quadriannulatus; ML = Anopheles melas; MR = Anopheles merus. CO, GA and H are defined based on SINE-PCR (Santolamazza et al., 2008). [file 12936_2014_522_MOESM1_ESM.docx]

**Table S1 - *Anopheles gambiae* complex sample for Intron-1 of VGSC gene sequencing data.**

| **Country** | **Locality** | **Year** | **Latitude/longitude** | ***N*** | ***Anopheles gambiae* complex** | | | | | | |
| --- | --- | --- | --- | --- | --- | --- | --- | --- | --- | --- | --- |
|  |  |  |  |  | ***GA*** | ***CO*** | **H** | ***AR*** | ***QD*** | ***ML*** | ***MR*** |
| Senegal | Kedougou | 2001 | 12°33'N, 12°10'W | 1 |  |  |  | 1 |  |  |  |
| The Gambia | Sare Samba Sowe | 2006 | 13°35'N, 15°1'W | 36 | 17 | 12 | 7 |  |  |  |  |
| Guinea Bissau | Antula | 2006 | 11°56'N, 15°35'W | 31 | 14 | 9 | 5 |  |  | 3 |  |
| Guinea Conakry | Sombili | 1990 | 11°24'N, 12°16'E | 2 | 2 |  |  |  |  |  |  |
| Mali | N'Gabacoro | 2000 | 12°41'N, 07°50'W | 1 |  | 1 |  |  |  |  |  |
| Burkina Faso | Dioulassouba | 2001 | 11°02'N, 04°13’W | 5 |  | 2 |  | 3 |  |  |  |
|  | Goundry | 1998 | 12°35'N, 01°02'W | 1 |  |  |  | 1 |  |  |  |
| Benin | Bohicon | 1998 | 07°10'N, 02°05'E | 1 |  | 1 |  |  |  |  |  |
| Nigeria | Kobape | 2001 | 07°00'N, 03°00'E | 4 |  | 1 |  | 3 |  |  |  |
| C.A.R. | Bayanga | 2001 | 02°53'N, 16°19'E | 2 |  | 2 |  |  |  |  |  |
| Cameroon | Mangoum | 1999 | 05°31'N, 10º37'E | 2 | 1 | 1 |  |  |  |  |  |
|  | Obala | 1999 | 04°09'N, 11º13'E | 1 | 1 |  |  |  |  |  |  |
| Angola | Luanda | 2000 | 08°45'S, 13°23'E | 3 | 1 |  |  |  |  | 2 |  |
| Kenya | Mbita | 2003 | 0°38'S, 37°36'E | 2 |  |  |  | 2 |  |  |  |
| Tanzania | Kilimani | 2009 | 05°16'S, 39°43'E | 2 |  |  |  |  |  |  | 2 |
| Rwanda | Mashesha | 2006 | 02°35'S, 29°00'E | 18 | 8 |  |  | 10 |  |  |  |
| Zimbabwe | Mana Pools Natl. Park | 2005 | 16°00'S, 28°34'E | 5 |  |  |  | 4 | 1 |  |  |
| ***Total:*** | | | | **117** | **44** | **29** | **12** | **24** | **1** | **5** | **2** |
