# Supplementary material for: Remarkable diversity of intron-1 of the para voltage-gated sodium channel gene in an Anopheles gambiae/Anopheles coluzzii hybrid zone
Source: Malar J. 2015 Jan 21;14:9. doi: 10.1186/s12936-014-0522-1 (PMC4308935; doi:10.1186/s12936-014-0522-1)
Supplement: Additional file 2: Table S2. — Intron-1 genotypes in Anopheles coluzzii and Anopheles gambiae (and putative hybrids) sampled in this study. N = number of genotyped individuals; Int-1702 = intron-1 genotype at position 702 (following Gentile et al., 2004); kdr = knock-down mutation at residue 1014:§ TTA = 1014 L (wild type); TTT = 1014 F (kdr-West); TCA = 1014S (kdr-East). [file 12936_2014_522_MOESM2_ESM.docx]

**Table S2 - Intron-1 genotypes in *Anopheles coluzzii* and *A. gambiae* (and putative hybrids) sampled in this study**.

| **Country** | ***N*** | ***Int-1 genotype*** | ***Int-1^702^*** | ***kdr^§^*** |
| --- | --- | --- | --- | --- |
|  |  |  |  |  |
| **Guinea Bissau** |  |  |  |  |
| *A. coluzzii* | 1 | M1/M1 | **C/C** | *TTA/TTA* |
|  | 1 | M3/M3 | **C/C** | *TTA/TTA* |
|  | 1 | M5/M5 | **C/C** | *TTA/TTA* |
|  | 1 | M1/M5 | **C/C** | *TTA/TTA* |
|  | 1 | M3/M5 | **C/C** | *TTA/TTA* |
|  | 1 | M1/GU1 | **C/C** | *TTA/TTA* |
|  | 1 | M3/GUGA | **C/C** | *TTA/TTA* |
|  | 1 | M3/GU3 | **C/C** | *TTA/TTA* |
|  | 1 | M3/GU4 | **C/C** | *TTA/TTA* |
| *A. gambiae* | 5 | S1/S1 | **T/T** | *TTA/TTA* |
|  | 2 | S1/M1 | **C/T** | *TTA/TTA* |
|  | 1 | S1/M3 | **C/T** | *TTA/TTA* |
|  | 1 | S1/GU6 | **C/T** | *TTA/TTA* |
|  | 1 | S1/GU7 | **T/T** | *TTA/TTA* |
|  | 1 | M1/GU2 | **C/C** | *TTA/TTA* |
|  | 3 | M3/GU5 | **C/T** | *TTA/TTA* |
| hybrids | 1 | S1/GU2 | **C/T** | *TTA/TTA* |
|  | 2 | S1/GU6 | **C/T** | *TTA/TTA* |
|  | 2 | M3/GUGA | **C/C** | *TTA/TTA* |
| **The Gambia** |  |  |  |  |
| *A. coluzzii* | 3 | M1/M1 | **C/C** | *TTA/TTA* |
|  | 1 | M3/M3 | **C/C** | *TTA/TTA* |
|  | 2 | M1/M3 | **C/C** | *TTA/TTA* |
|  | 1 | M1/GUGA | **C/C** | *TTA/TTA* |
|  | 5 | M3/GA3 | **C/C** | *TTA/TTA* |
| *A. gambiae* | 2 | S1/S1 | **T/T** | *TTA/TTA* |
|  | 1 | S1/S7 | **T/C** | *TTA/TTA* |
|  | 2 | S1/GA6 | **T/T** | *TTA/TTA* |
|  | 1 | S1/M1 | **C/T** | *TTA/TTA* |
|  | 2 | M3/M3 | **C/C** | *TTA/TTA* |
|  | 1 | M3/M1 | **C/C** | *TTA/TTA* |
|  | 1 | M1/GA6 | **C/T** | *TTA/TTA* |
|  | 2 | M3/GA3 | **C/C** | *TTA/TTA* |
|  | 4 | M3/GA4 | **C/T** | *TTA/TTA* |
|  | 1 | GA1/GA5 | **C/T** | *TTA/TTA* |
| hybrids | 3 | S1/S1 | **T/T** | *TTA/TTA* |
|  | 1 | M3/M3 | **C/C** | *TTA/TTA* |
|  | 1 | S1/M1 | **C/T** | *TTA/TTA* |
|  | 1 | M1/GA7 | **C/C** | *TTA/TTA* |
| **G. Conakry** |  |  |  |  |
| *A. gambiae* | 2 | S1/S1 | **T/T** | *TTA/TTA* |
| **Mali** |  |  |  |  |
| *A. coluzzii* | 1 | M1/M7 | **C/C** | *TTA/TTA* |
| **Burkina Faso** |  |  |  |  |
| *A. coluzzii* | 2 | M1/M7 | **C/C** | *TTA/TTA* |
| **Benin** |  |  |  |  |
| *A. coluzzii* | 1 | M5/S1 | **C/T** | *TTA/TTA* |
| **Nigeria** |  |  |  |  |
| *A. coluzzii* | 1 | M5/M7 | **C/C** | *TTA/TTA* |
| **G. Conakry** |  |  |  |  |
| *A. gambiae* | 2 | S1/S1 | **T/T** | *TTA/TTA* |
| **C.A.R.** |  |  |  |  |
| *A. coluzzii* | 1 | M1/M1 | **C/C** | *TTA/TTA* |
|  | 1 | M1/M8 | **C/C** | *TTA/TTA* |
| **Cameroon** |  |  |  |  |
| *A. coluzzii* | 1 | M1/M1 | **C/C** | *TTA/TTA* |
| *A. gambiae* | 1 | S1/S9 | **T/T** | *TTA/TTA* |
|  | 1 | S1/S10 | **T/T** | *TTA/TTA* |
| **Angola** |  |  |  |  |
| *A. gambiae* | 1 | S1/S8 | **T/T** | *TTA/TTA* |
| **Rwanda** | 1 | S1/S1 | **T/T** |  |
| *A. gambiae* |  |  |  | *TCA/TCA* |
|  | 1 | M1/S1 | **C/T** | *TCA/TCA* |
|  | 2 | M1/S1 | **C/T** | *TTA/TTA* |
|  | 1 | M1/S1 | **C/T** | *TTT/TTA* |
|  | 1 | M1/M1 | **C/C** | *TTT/TTA* |
|  | 1 | S7/S1 | **C/T** | *TTA/TTA* |
|  | 1 | S6/M1 | **C/T** | *TTT/TTA* |
